# Supplementary material for: Global hypo-methylation in a proportion of glioblastoma enriched for an astrocytic signature is associated with increased invasion and altered immune landscape
Source: eLife. 2022 Nov 22;11:e77335. doi: 10.7554/eLife.77335 (PMC9681209; doi:10.7554/eLife.77335)
Supplement: Figure 2—source data 1. [file elife-77335-fig2-data1.zip › Figure_2_source_data_1/Figure_2B/homerResults/motif76.similar.html]

motif76

## Information for motif76

T
C
A
G
C
T
A
G
C
T
A
G
T
C
A
G
C
T
G
A
T
C
A
G
T
C
A
G
T
C
A
G
C
T
A
G
C
T
A
G
C
T
A
G
T
C
A
G
C
T
A
G
T
C
A
G
C
T
A
G
T
C
A
G
C
T
A
G
C
T
A
G
T
C
A
G
T
C
A
G
C
T
A
G
T
C
A
G
T
C
A
G
T
C
A
G
T
C
A
G
  
Reverse Opposite:  

A
G
T
C
A
G
T
C
A
G
T
C
A
G
T
C
G
A
T
C
A
G
C
T
A
G
T
C
G
A
T
C
G
A
T
C
A
G
T
C
A
G
T
C
A
G
T
C
G
A
T
C
A
G
T
C
G
A
T
C
G
A
T
C
G
A
T
C
A
G
T
C
A
G
T
C
A
G
T
C
G
A
C
T
A
G
T
C
G
A
T
C
G
A
C
T
A
G
T
C
  

|  |  |
| --- | --- |
| p-value: | 1e-7 |
| log p-value: | -1.658e+01 |
| Information Content per bp: | 1.321 |
| Number of Target Sequences with motif | 901.0 |
| Percentage of Target Sequences with motif | 38.99% |
| Number of Background Sequences with motif | 119.9 |
| Percentage of Background Sequences with motif | 33.92% |
| Average Position of motif in Targets | 98.0 +/- 50.2bp |
| Average Position of motif in Background | 97.2 +/- 52.7bp |
| Strand Bias (log2 ratio + to - strand density) | -0.1 |
| Multiplicity (# of sites on avg that occur together) | 1.31 |
| Motif File: | file (matrix) reverse opposite |

### Similar de novo motifs found

|  |  |  |  |  |  |  |  |
| --- | --- | --- | --- | --- | --- | --- | --- |
| Rank | Match Score | Redundant Motif | P-value | log P-value | % of Targets | % of Background | Motif file |
| 1 | 0.899 | A G T C A G T C G A T C A G T C A G T C G A T C G A T C A G T C A G T C G A T C G A T C A G T C G A T C G A T C A G T C G A T C G A T C G A T C G A T C G A T C | 1e-3 | -7.694874 | 0.74% | 0.26% | motif file (matrix) |
| 2 | 0.664 | A G T C A G T C A G T C G A C T A G T C A G C T A G T C A G T C A G T C A G T C A G T C A G T C A G T C A G T C A G T C A G T C A G T C A G T C A G T C A G T C A G T C A G T C A G T C A G T C A G T C A G C T A G T C A G T C A G T C A G T C A G T C A G T C A G T C A G T C A G T C G A T C A G T C A G T C A G T C A G T C A G T C A G T C G A T C G A C T A G T C A G T C A G T C G A T C A G T C G A T C | 1e-2 | -5.058320 | 20.47% | 18.58% | motif file (matrix) |
| 3 | 0.866 | A G T C A G T C A G T C A G T C A G T C A G T C A G T C A G T C A G T C A G T C A G T C A G T C A G T C A G T C A G T C A G T C A G T C A G T C A G T C A G T C A G T C A G T C A G T C A G T C A G T C A G T C A G T C A G T C A G T C A G T C | 1e-1 | -4.220897 | 43.62% | 41.33% | motif file (matrix) |
